# A microenvironment prediction model for Chinese solar greenhouses based on the bond graph approach

**DOI:** 10.1371/journal.pone.0267481

**Published:** 2022-05-03

**Authors:** Lei Zhang, Xingan Liu, Tianlai Li, Jianwei Ji, Lei Zhao

**Affiliations:** 1 College of Information and Electrical Engineering, Shenyang Agricultural University, Shenhe District, Shenyang, China; 2 National & Local Joint Engineering Research Center of Northern Horticultural Facilities Design & Application Technology (Liaoning), Shenhe District, Shenyang, China; 3 Key Laboratory of Protected Horticulture, Shenyang Agricultural University, Ministry of Education, Shenhe District, Shenyang, China; 4 College of Horticulture, Shenyang Agricultural University, Shenhe District, Shenyang, China; 5 Liaoning Petrochemical University, College of Petroleum Engineering, Wanghua District, Fushun, Liaoning Province, China; Tongji University, CHINA

## Abstract

To improve the prediction accuracy of temperature and humidity in typical Chinese solar greenhouses, this paper proposed a new longwave/shortwave radiation modeling method using bond graph. This model takes into account sun position, useful incoming solar radiation model, sky longwave radiation model, inside longwave, and shortwave radiation model. The approach solves the problems caused by underestimating the effects of longwave radiation on night temperature and relative humidity. The study found that after a period of t = 7.5 h, with the increase of sun altitude angle, the internal temperature was significantly affected by the temperature rise of outside environment on sunny day. The sun altitude angle gradually falls over a period of t = 12.5 h (beginning at 12.30 p.m.). The decline in night temperature steadily slowed after a period of t = 20.5 h. On the other hand, the temperature variation has a multi-peak distribution and the warming rate of the CSG slows down on cloudy days. Furthermore, a good agreement between the experimental and simulation data were obtained, with a maximum temperature deviation of 2°C and maximum humidity deviation of 5%. The developed model is a universal and valuable approach that can be used for greenhouse climate simulation. Furthermore, it can be used as a support system during decision-making processes to help manage Chinese solar greenhouses more efficiently, which provides several control perspectives on the low-energy greenhouse in the future. This work has also provided several control perspectives on the low energy greenhouse in the future.

## Introduction

In most countries, the greenhouse agricultural sector represents the largest consumer of total energy [1]. Chinese solar greenhouse (CSG) is an important agricultural facility for the cultivation of vegetables and fruits in cold regions throughout the year [2]. Moreover, it plays an important role in winter cultivation of agricultural products in northern China. According to 2021 statistics, solar greenhouses in China covered up to 1.96 million ha [3], which corresponds to 30% of the total horticultural areas in this country. The CSG can achieve high-efficiency energy utilization without any auxiliary heating. What has it done? It is because the enclosing body of the CSG, especially the north wall (i.e. back wall), has the capacities of heat storage-release and thermal preservation. The north wall of CSG can maintain the temperature and humidity at an appropriate level to create a suitable environment for crop growth. By using phase change material (PCM) we could even improve the heat storage capacity. So the CSG construction can receive the solar radiation energy to realize self-sufficiency and reduce the cost of artificial heating [4].

Mobtaker et al. [5] investigated six shapes of greenhouses including: uneven span, even span, single span, vinery, quonset and arch type from solar radiation availability point of view. In general, conventional experiments are expensive, inaccurate, and display low levels of environmental performance and predictability [5, 6]. However, CSG modeling provides functionality, portability, and applicability, which allows understanding the effects of various parameters on cooling/heating demand, as well as to obtain optimal operating conditions. It is of fundamental importance for proper selection of greenhouse design parameters and for decision-making purposes during production [7]. Depending on conventional model, designers have to select the features that provide the desired characteristics and identify the optimal model [8]. In CSG, the environmental prediction models are classified into two types: (a) those based on heterogeneous approaches using computational fluid dynamics (CFD) codes; and (b) homogeneous, based on heat and mass balance equations. In the first approach, variables are described through spatial coordinates inside the greenhouse. In this respect, Guzman et al. [9] combined CFD analysis and a control strategy to obtain system models for monitoring the temperature in greenhouses. He et al. [10] investigated the effect of vent dimension on greenhouse cooling in a solar greenhouse with CFD. Piscia et al. [11] presented a CFD greenhouse night-time condensation model. The results showed the importance of heat transfer losses by radiation, particularly for low values of soil heat flux. Amara et al. [12] analyzed the effect of the shading induced by south oriented photovoltaic panels on the distributed climate and plant activities in a mono-span greenhouse by using CFD approach. The second approach [13, 14] considers that temperature and humidity are homogenous in different sections of the greenhouse. Energy and mass balances allow treating temperature and humidity in a lumped way [8, 13]. It is important to consider that the external environment affects the internal greenhouse air. Baglivo et al. [15] considered simultaneously different thermal phenomena with detailed modeling including dense volume discretization, 3D shortwave and longwave radiative exchange, air flow exchanges, presence of lamps with their exact 3D position, ground and plant evapotranspiration, and convective heat transfer coefficients.

Both advantages and disadvantages of the two methods were identified. CFD codes are more suitable for modeling complex flows. Unfortunately, it requires long execution times and high computational resources. Different control devices have been applied to CSG, including climate sensors [16], artificial light [17], CO^2^ enrichment [18], and fan cooling systems [19], among others. The control engineers [8, 20] usually rely on lumped parameter models, which are useful for generating low order linear models and for initial validation of the closed loop control. Ali et al. [21] designed a fuzzy logic controller for insulated greenhouse (IG) with the exploitation of wind energy in order to minimize energy consumption. Bonuso et al. [22] focuses on the ventilation analysis of solar greenhouse with symmetrical flat pitched roof and single span located in a warm temperate climate. Zhang et al. [23] presented a method to evaluate micro-light climate and thermal performance on a CSG. They included a detailed 3D tomato canopy structure, which was simulated using a functional–structural plant model. However, this method did not consider the effect of wavelength.

In the last decades, a significant amount of research has been devoted to solar radiation [24, 25]. However, its effect on CSG has not been included. The goal of the present study is to develop a model that provides information about how solar radiation affects CSG microclimate. We intend to describe complex systems in a concise manner, and improve the effectiveness of complex situations. Bond graph is a feasible method widely used to describe the energy structure of systems [26, 27], and an effective modeling tool for dynamic systems simulation. Abbes et al. applied the bond graph model to plastic greenhouses and heating systems in the Northern Hemisphere [28, 29]. Su et al. [30] presented a bond graph model including floor, concrete, heat exchanger and soil. Bot et al. [31] published a greenhouse bond graph model without considering the impact of wavelength. However, traditional bond graph modeling cannot be directly applied to CSG because the enclosing body of the solar greenhouse. The CSG receives the proper amount of solar radiation that is needed for a self-sufficient facility, reducing the cost of artificial heating. Nevertheless, sunny and cloudy days have a different effect on greenhouse climate. The amount of longwave solar radiation depends on day-night conditions and the presence of clouds. Thus, a thorough understanding of the effects of wavelength on the greenhouse climate response and related mechanisms is required.

In the present research, a complete homogeneous CSG model was obtained using the bond graph theory. This model is simple and efficient for predicting temperature and humidity in the CSG. On the CSG bong graph model, the present research aims to create a full longwave and shortwave radiation model, which solves the problem of underestimating the influence of longwave radiation on night temperature and humidity. The sky longwave radiation flux, which is affected by diurnal/nocturnal conditions and cloud cover rate, is given special consideration in the CSG. Furthermore, the flexibility of bond graph allows the implementation of a radiation model directly on the bond graph model while taking physical specifications into account, such as the impact of sun radiation on temperature and relative humidity at different greenhouse locations. Several constitutive equations were integrated into the model in order to determine the mechanisms and processes that occur in a real CSG. Moreover, the data provide important theoretical basis for the proper analysis of energy flow and environmental control. In practical engineering application, it can help improve the growing environment of crops. Therefore, it has reference significance for the study of temperature and humidity control system of solar greenhouse in the future.

## Experiments and instruments

**Fig 1:**
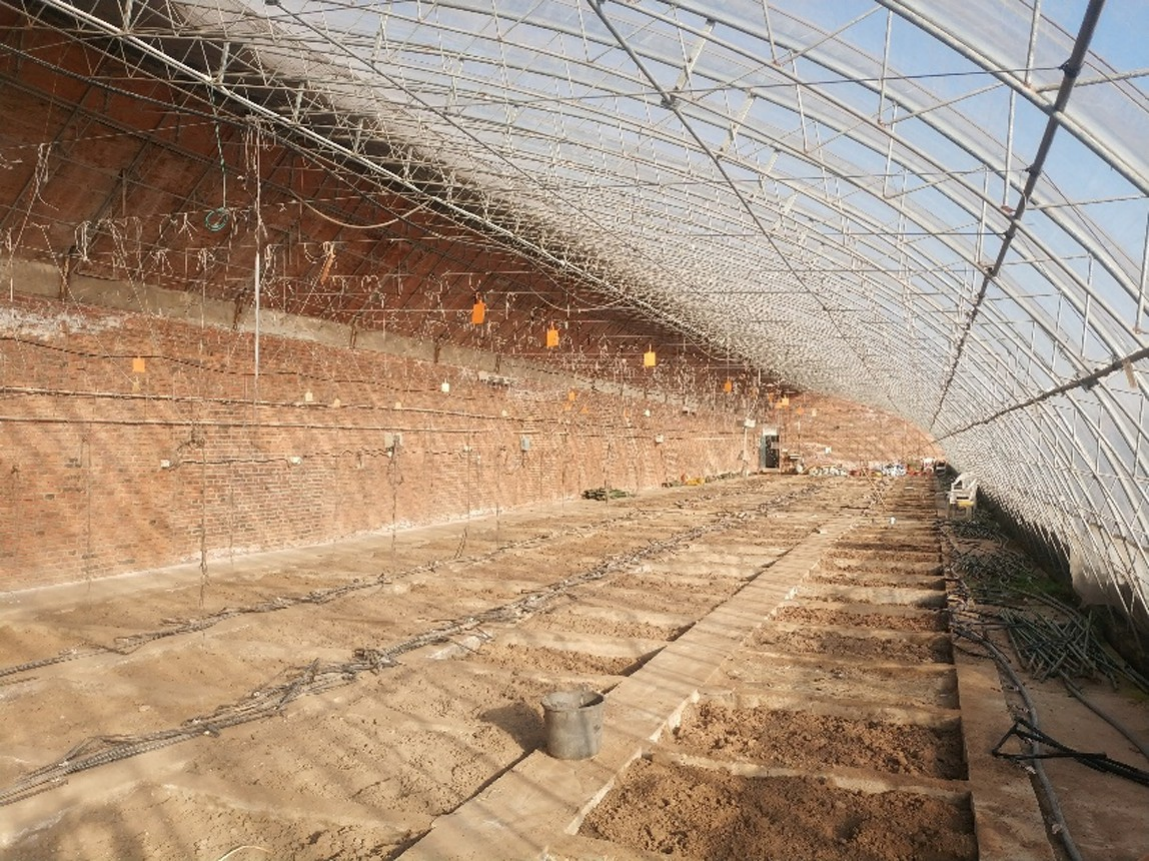
Structure of the experimental Chinese solar greenhouse schematic diagram.

**Fig 2:**
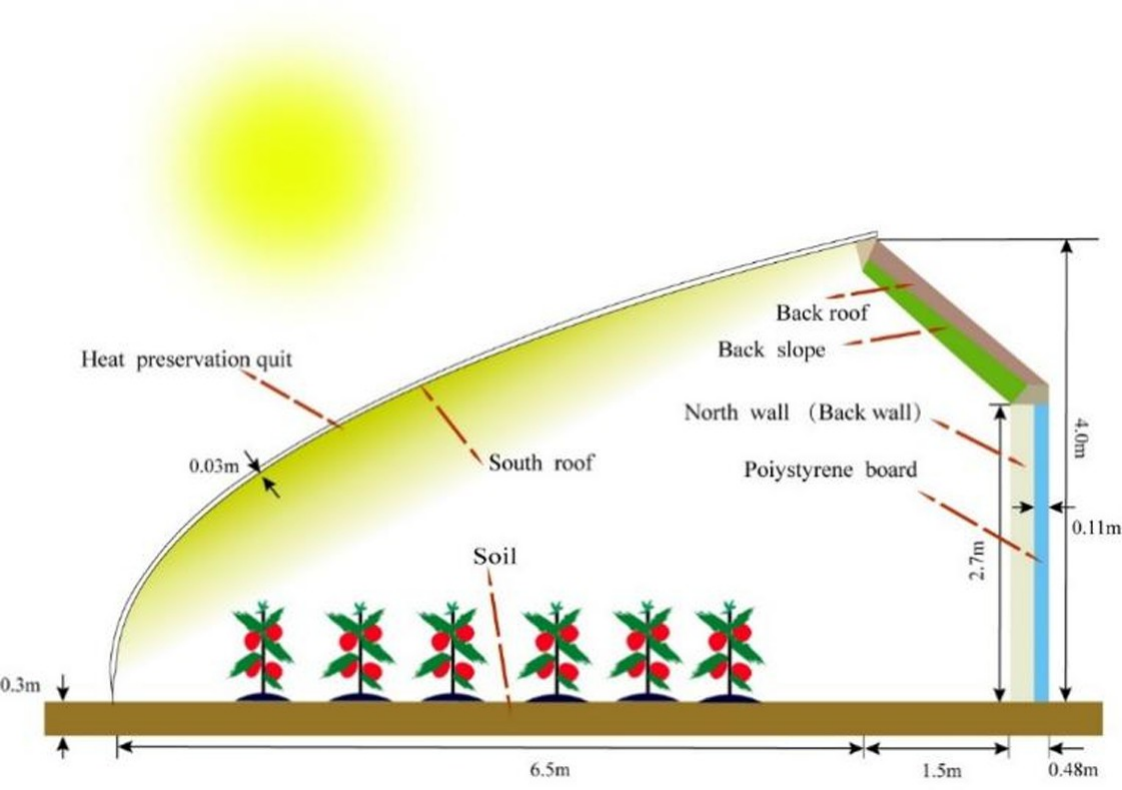
Schematic diagram of the Chinese solar greenhouse.

The model validation was carried out using the CSG presented in Fig 1. The CSG was located at 41°49′N, 123°34′E, oriented north-south, and the solar azimuth angle was of 7° from south to west. Moreover, the CSG displayed a ridge height of 4m, a north wall height of 2.7m, a span of 8m, and a length of 60 m. As shown in Fig 2, the roof structure was composed of steel trusses with no pillars inside. The external structure of the north wall was made of polystyrene board and a fine coal ash brick wall was selected as internal structure.

**Fig 3:**
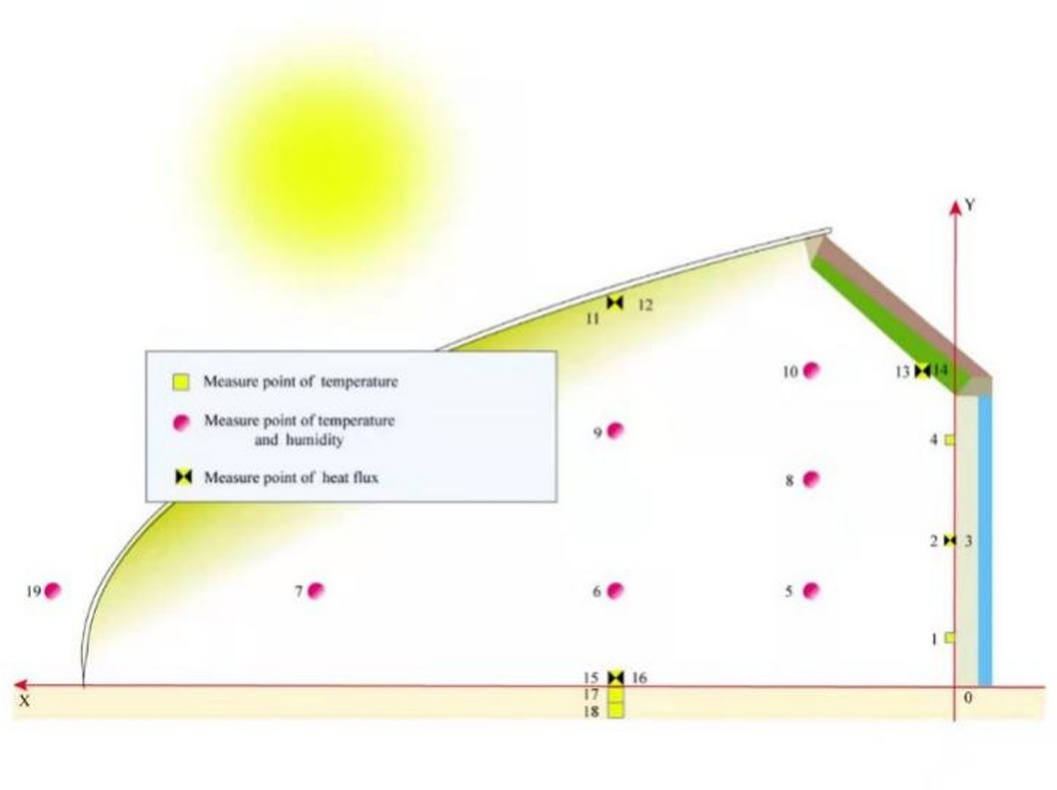
Layout of temperature and humidity sensors.

The internal structure of the CSG is shown in Fig 3. In our experiments, we selected PO as the plastic film, and the heat insulation quilt was made of three layers of composite materials. The polystyrene board is an excellent thermal insulation material, which displays low heat-transfer coefficient (just 0.041 W/mK), and low density (just 18 kg/m³). At present, polystyrene board is widely used as the external thermal insulation composite system in the north wall of CSG. The temperature and humidity in the CSG were recorded using a HOBO recorder. Data at a given position was recorded at one-minute intervals and the collected data were preserved as text. The temperature probe was provided with a radiation shield and presented a standard accuracy of ±0.2°C. The temperature measuring instrument also included a T-type thermocouple. CR1000 and CR3000 data collector produced by Campbell Company were used for data acquisition. The thermocouples were used to measure the surface temperature of the wall and the air temperature was also shielded. A HFP01 sensor produced by Hukseflux Company and containing a ceramic and plastic material shell was used to measure the heat flux in soil, walls, and other parts of the building. The regular voltage signals were transmitted to the surroundings in order to collect heat flux data.

## Radiative transfer schemes and model assumptions

The CSG can be divided into six sections: cover, internal air, vegetation, soil, back slope, and north wall. Each field interacts with its neighbor by means of heat and mass transfer, or radiation. The main physical processes driving the energy flow in the greenhouse include short and long wave radiation exchange, heat and mass convection heat transfer, soil conduction and convection heat transfer, air heat and mass transfer through the roof, back slope and internal air convection exchange, and north wall and internal air convection exchange.

Before building the CSG model, some assumptions were included. The secondary factors were disregarded. The interior and exterior of the CSG was considered homogeneous and isotropic, at a steady-state flow, and the gas mixture was assumed to conform to the ideal gas law. Heat and mass transfer around the canopy film, vegetation, soil, back slope, and north wall obeyed the boundary layer theory. All surfaces involved in radiative transfer were regarded as grey surfaces. The direct radiation in the greenhouse was neglected because it is mostly diffused. The direction of the connections was determined by assuming that the energy transfer from the deep soil to the external environment was positive. In addition, considering the boundary layer theory, boundary layer 1 was set between soil and air; boundary layer 2 was the boundary layer between air and vegetation; and boundary layer 3 was that between the air and greenhouse membrane. These three areas were selected by considering greenhouse assumptions previously published [29].

## Bond graph model of the greenhouse system

**Fig 4:**
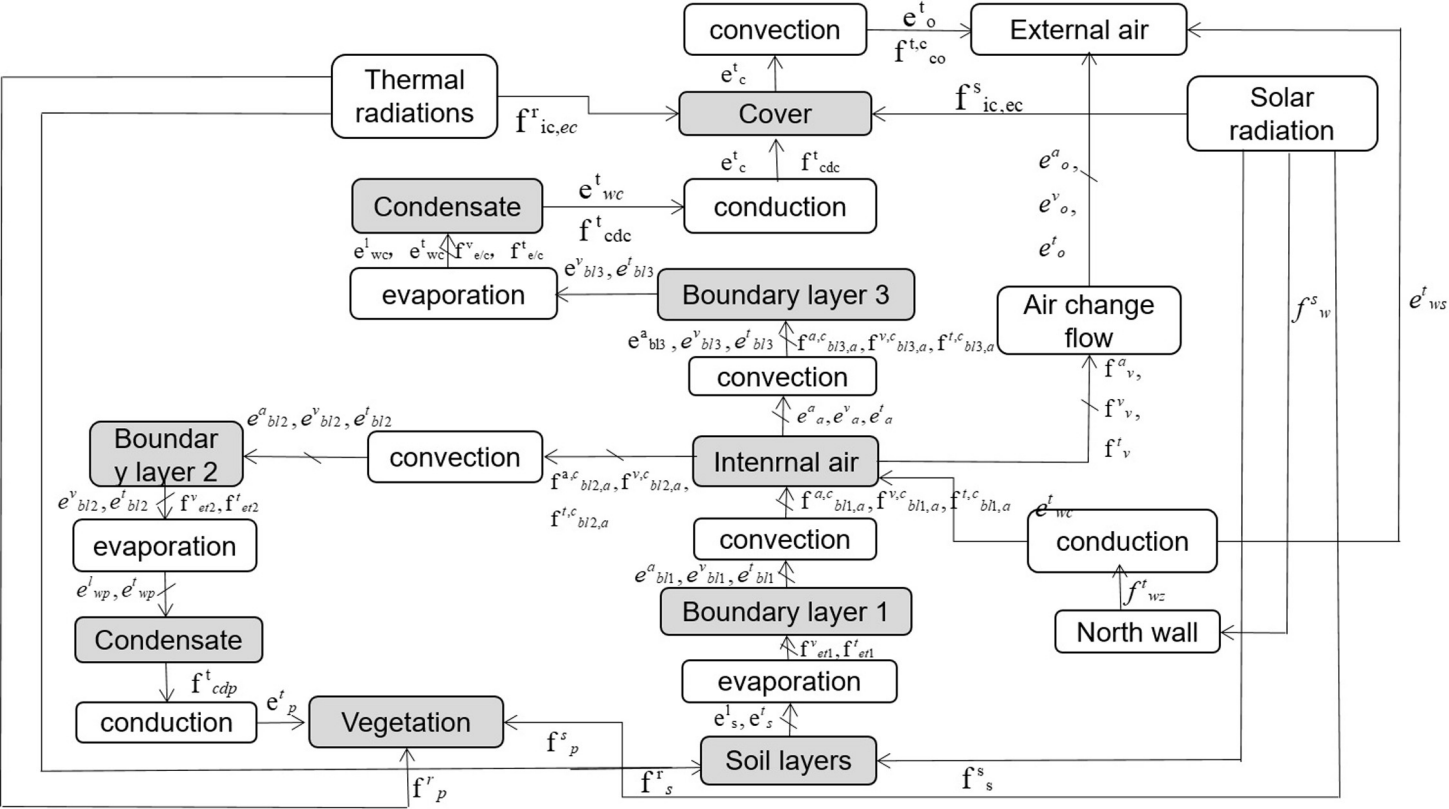
Energy structure of the CSG system.

For solar radiation and thermal radiation bonding, temperature flow and pressure flow were considered as potential variables. Radiation flow and heat flow were considered as flow variables. The bonding diagram is shown in Fig 4. Thermal radiation and solar radiation were modeled. In addition, the heat flow directly generated by solar and thermal radiation entered the areas in contact with sunlight (i.e. cover, vegetation, soil, back slope, and north wall), and the real radiation conditions were simulated using the bonding graph method. Solar radiation determines the radiation flux reaching each element of the greenhouse. Thus, we firstly performed a direct/diffuse separation. Secondly, the incoming radiation stream was determined from direct solar radiation by considering the position of the sun. Subsequently, the solar radiation flow was solved by considering the influence of the membrane geometry. Finally, the radiation flow that reached the greenhouse was determined according to the radiation flow transmitted through the south roof.

**Fig 5:**
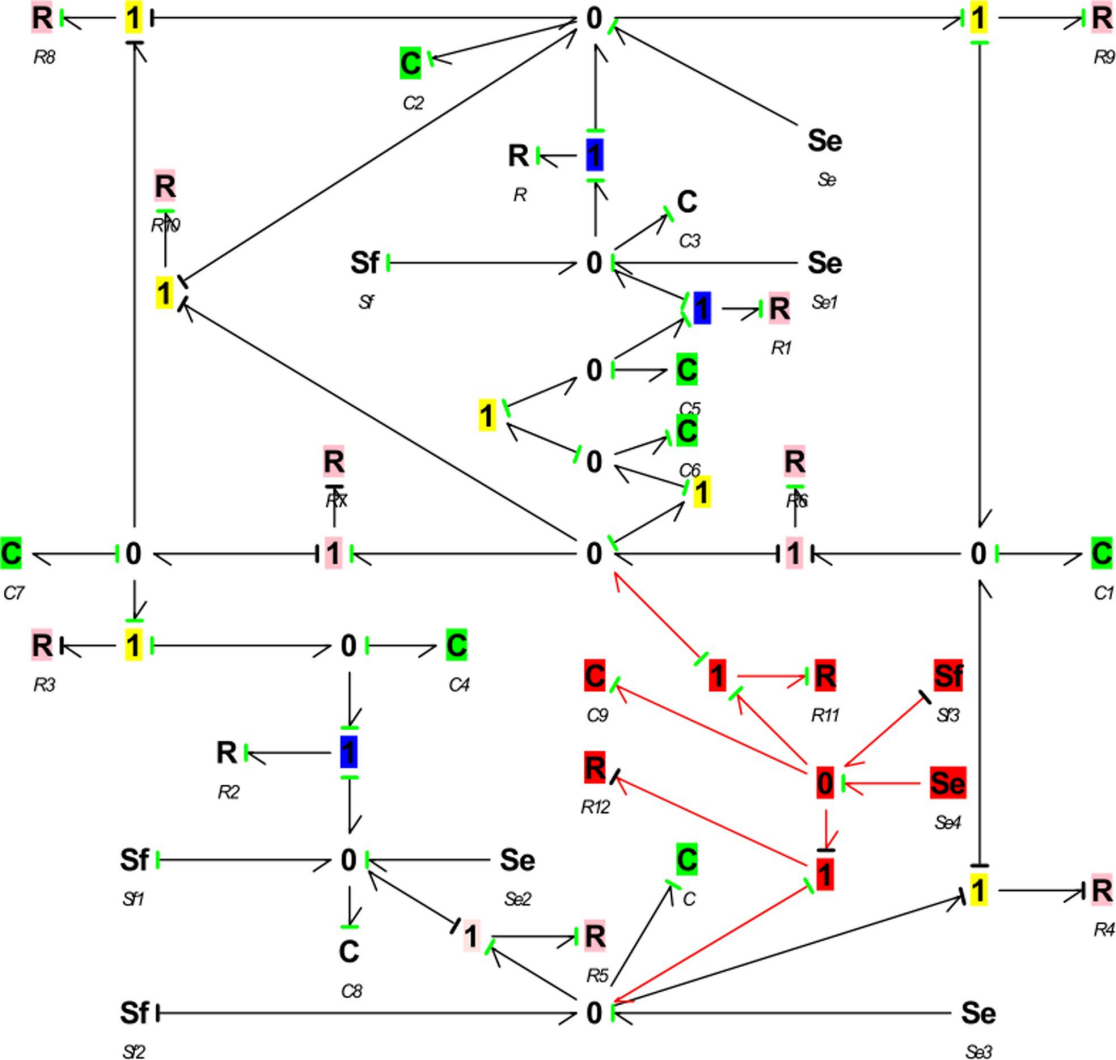
Bond diagram model of CSG.

Thermal radiation block calculates the heat flow produced by thermal radiation of greenhouse components. The parameters were determined considering heat flow inside and outside the greenhouse. Fig 5 presents the bonding diagram model of the CSG. Sf element is a direct representation of radiation heat flow in the actual parts of the solar greenhouse (i.e. soil, vegetation, canopy membrane, back slope, and north wall).

## Mathematical CSG model

The R and C elements compute flow and potential functions, respectively, by using Eq (1) [30, 31]:

$$e^{i}=\phi_{c}\left(\int f^{i}dt\right),f^{i}=\phi_{R}^{-1}\left(e^{i}\right)$$ (1)

where *ϕ*_*R*_ and *ϕ*_*c*_ depend on the geometric conditions and thermal physical parameters of the model; *e*^*i*^ represents the potential function; and *f*^*i*^ corresponds to the flow function. Se and Sf, which are known variables of the model, represent the potential source and flow source, respectively. Flow source indicates heat flow caused by thermal radiation, and potential source is heat flow caused by solar radiation. These variables correspond to known data from the bond graph model, but they are predetermined by solar and thermal radiation. The parameters related to thermal radiation and solar radiation models are shown in Table 1, as well as the thermo-physical parameters and constants.

**Table 1:** Parameters used for the computation.

Parameter	Value	Unit	Source	Parameter	Value	Unit	Source
*a* ^*a*^	214.7	10^−7^ *m*^2^ · *s*^*a*-1^	Baehr [32]	*a*_*s*_	0.8	—	Cabrera [33]
*a* ^*v*^	3283	10^−7^ *m*^2^ · *s*^*a*-1^	Baehr [32]	$\varepsilon_{C}^{*}$	0.9	—	Mahdouri [6]
*A*_*C*_	199.4	*m* ^2^	constant	$\varepsilon_{es}^{*}$	0.9	—	Cabrera [33]
*A*_*f*_	23.8	*m* ^2^	constant	$\varepsilon_{p}^{*}$	0.96	—	Cabrera [33]
*A*_*ρ*_	200	*m* ^2^	constant	$\varepsilon_{s}^{*}$	0.9	—	Cabrera [33]
*A*_*s*_	100	*m* ^2^	constant	*V*_*a*_	220	*m* ^3^	constant
*CP* ^*a*^	1007	*J* · *kg*^1^ · *K*^1^	Lide [26]	θ*	0.402	—	constant
*CP* ^*l*^	4181	0.43	Lide [26]	*θ*_*dry*_	0.43	—	constant
*CP* ^*v*^	1892	*J* · *kg*^1^ · *K*^1^	Lide [26]	*λ*	10.22	0	constant
*CP*_*p*_	2500	0.02569	Cabrera [33]	λ*	0.93	*W* · *m*^−2^ · *K*^−1^	Lide [26]
*CP*_*c*_	75.1	*J* · *m*^−3^ · *K*^1^	Cabrera [33]	*λ* ^*a*^	0.0257	*W* · *m*^−2^ · *K*^−1^	Baehr [32]
D	0.292	10^−4^ *m*^2^ · *s*^-1^	Baehr [32]	*λ* ^*c*^	0.33	*W* · *m*^−2^ · *K*^−1^	constant
*F*_*cc*_	0.49862	—	Cabrera [33]	*λ*_*dry*_	0.14	*W* · *m*^−2^ · *K*^−1^	Baehr [32]
*F*_*cp*_	0.13738	—	Cabrera [33]	*λ* ^*p*^	0.5	*W* · *m*^−2^ · *K*^−1^	Cabrera [13]
*F*_*cs*_	0.31888	—	Cabrera [33]	*λ* ^*v*^	0.0189	*W* · *m*^−2^ · *K*^−1^	Lide [26]
*F*_*cse*_	0.296	—	Cabrera [33]	*λ*_*z*_	15	0	—
*F*_*c∞*_	0.704	—	Cabrera [33]	*ρ* ^a^	1.188	*kg* · *m*^−3^	Baehr [32]
*F*_*pc*_	0.4775	—	Cabrera [33]	*ρ* ^*l*^	998.21	*kg* · *m*^−3^	Baehr [32]
*F*_*pp*_	0.067504	—	Cabrera [33]	*ρ* ^s^	2650	*kg* · *m*^−3^	—
*F*_*ps*_	0.455	—	Cabrera [33]	*ρ* ^v^	0.02659	*kg* · *m*^−3^	Baehr [32]
*F*_*sec*_	0.02	—	Cabrera [33]	*ρ*_c_	0.1	—	Cabrera [33]
*F*_*se∞*_	0.98	—	Cabrera [33]	$\rho_{c}^{*}$	0.07	—	Cabrera [33]
*F*_*sc*_	0.636	—	Cabrera [33]	$\rho_{es}^{*}$	0.1	—	Cabrera [33]
*F*_*sp*_	0.364	—	Cabrera [33]	*ρ*_p_	0.6	—	Cabrera [33]
*G*_*sc*_	1367	*W* · *m*^−2^	He [10]	$\rho_{p}^{*}$	0.04	—	Cabrera [33]
Kh*	0.000005	*M* · *s*	constant	*ρ*_s_	0.2	—	Cabrera [33]
*cp*	2540	*KJ* · *kg*^−1^	constant	$\rho_{s}^{*}$	0.1	—	Cabrera [33]
*LAI*	4	—	constant	*σ*	5.6705 10^−8^	*W* · *m*^−2^ · *K*^4^	constant
${\tilde{M}}^{w}$	0.01802	*Kg* · *mol*^−1^	constant	*τ*_*c*_	0.7	—	Mashonjowa [34]
${\tilde{M}}^{a}$	0.02896	*Kg* · *mol*^−1^	constant	$\tau_{c}^{*}$	0.77	—	Cabrera [33]
*a*_*ec*_	0.2	—	Mashonjowa [34]	*ϕ*	36	0	Liu [35]
*a*_*ic*_	0.1	—	Cabrera [27]	*C*	0.85	w*m*^−2^ · *K*^−1^	Liu [35]
*a*_*p*_	0.4	—	Cabrera [33]				

The conservation law of each field can be determined at zero:


$$\text{Cover}\;0_{1}:\;f_{c}^{t}=f_{cdc}^{t}-f_{co}^{t,c}+f_{ic}^{s}+f_{ec}^{s}+f_{ic}^{r}+f_{ec}^{r}$$ (2)

$$\text{Vegetation}\;0_{2}:\;f_{p}^{s}=f_{cdp}^{t}+f_{p}^{t}+f_{p}^{r}$$ (3)

$$\text{Soil}\;\text{Surface}\;0_{3}:\;\left\{\begin{array}{c} f_{s}^{t}=f_{1s}^{t}-f_{et1}^{t}+f_{s}^{s}+f_{s}^{r}\\ f_{s}^{l}=f_{1s}^{l}-f_{et1}^{l} \end{array}\right.$$ (4)

$$\text{Internal}\;\text{air}\;0_{4}:\;\left\{\begin{array}{c} f_{a}^{t}=f_{bl1,a}^{t,c}-f_{a,bl2}^{t,c}-f_{a,bl3}^{t,c}-f_{v}^{t}\\ f_{a}^{v}=f_{bl1,a}^{v,c}-f_{a,bl2}^{v,c}-f_{a,bl3}^{v,c}-f_{v}^{v}\\ f_{a}^{a}=f_{bl1,a}^{a,c}-f_{a,bl2}^{a,c}-f_{a,bl3}^{v,c}-f_{v}^{a} \end{array}\right.$$ (5)

$$\text{Boundary}\;\text{Layer}\;1\;0_{5}:\;\left\{\begin{array}{c} f_{bl1}^{t}=f_{bl1,a}^{t,c}-f_{et1}^{t}\\ f_{bl1}^{v}=f_{bl1,a}^{v,c}-f_{et1}^{v}\\ f_{bl1}^{a}=f_{bl1,a}^{a,c} \end{array}\right.$$ (6)

$$\text{Boundary}\;\text{Layer}\;2\;0_{6}:\;\left\{\begin{array}{c} f_{bl2}^{t}=f_{bl2,a}^{t,c}-f_{et2}^{t}\\ f_{bl2}^{v}=f_{bl2,a}^{v,c}-f_{et2}^{v}\\ f_{bl2}^{a}=f_{bl2,a}^{a,c} \end{array}\right.$$ (7)

$$\text{Boundary}\;\text{Layer}\;3\;0_{7}:\;\left\{\begin{array}{c} f_{bl3}^{t}=f_{bl3,a}^{t,c}-f_{e/c}^{t}\\ f_{bl3}^{v}=f_{bl1,a}^{v,c}-f_{e/c}^{v}\\ f_{bl3}^{a}=f_{bl3,a}^{a,c} \end{array}\right.$$ (8)

$$\text{Air}\;\text{condensed}\;\text{air}\;\text{on}\;\text{vegetation}\;0_{8}:\;\left\{\begin{array}{c} f_{wp}^{t}=f_{et2}^{t}-f_{cdp}^{t}\\ f_{wp}^{l}=f_{et2}^{v} \end{array}\right.$$ (9)

$$\text{Air}\;\text{condensed}\;\text{on}\;\text{the}\;\text{membrane}\;0_{9}:\;\left\{\begin{array}{c} f_{wc}^{t}=f_{e/c}^{t}-f_{cdc}^{t}\\ f_{wc}^{l}=f_{e/c}^{v} \end{array}\right.$$ (10)

Among them

$$\begin{array}{l} f_{ic}^{s}=\alpha_{ic}A_{c}E_{ic},f_{ec}^{s}=\alpha_{ec}A_{c}E_{ec},f_{p}^{s}=\alpha_{p}A_{p}E_{p},f_{s}^{s}=\alpha_{s}A_{s}E_{s}\\ f_{ic}^{r}=A_{c}q_{ic},f_{ec}^{r}=A_{c}q_{ic},f_{p}^{r}=A_{p}q_{p},f_{s}^{r}=A_{s}q_{s} \end{array}$$ (11)

The heat flux generated by solar radiation depends on several parameters including solar irradiation, solar position, local time, and geographical location (i.e. latitude and longitude). As is known, modeling solar radiation in CSG is very important. Thus, in order to improve the accuracy, modeling was performed through the following steps:

When the horizontal surface at the outer atmosphere is considered, the effective incident solar radiation represents the normal component of solar radiation on that surface. This is denoted by

$$G_{0}=G_{sc}\left(1+0.033\;cos\frac{2\pi n}{365}\right)\left(cos\;\delta \;cos\;w+sin\;\delta \;sin\;\phi \right)$$ (12)

$$\delta =23.45\;sin\left(2\pi \frac{284+n}{365}\right)\;and\;w=15\times \left(\frac{t}{3600}-12\right)$$ (13)

where *ϕ* is latitude; *ω* corresponds to hour Angle; *t* stands for time in base 24 (s); *δ* is declination; *n* indicates a given day of the year; and *G*_*SC*_ is a solar constant [25].

When solar radiation enters the atmosphere and reaches the soil surface, it is divided into direct and diffuse components, as shown in Eq (14)

$$G_{t}=G_{D}+G_{d}$$ (14)

where *G*_*t*_ represents solar radiation; *G*_*D*_ is the direct radiation; and *G*_*d*_ indicates diffuse radiation. Solar radiation can be divided into direct radiation and diffuse radiation. These parameters are employed to estimate the flux of solar radiation heat reaching the greenhouse film. Because the data of the solar heliometer deals with diffuse and direct solar radiation, direct and diffuse radiation should be separated. The conventional method to perform this is to find out the correlation between *G*_*D*_ and *G*_*d*_, and *G*_*t*_ and sky clearness index *kt* [26]. The sky clearness index is calculated using Eq (15):

$$k_{t}=\frac{G_{t}}{G_{0}}$$ (15)

These correlations are based on measurements of global and diffuse irradiance on horizontal planes. According to the model proposed by Louche et al. [36], the resulting correlation is shown in Eq (16):

$$\frac{G_{D}}{G_{0}}=-10.627k_{t}^{5}+15.307k_{t}^{4}-5.205k_{t}^{3}+0.994k_{t}^{2}-0.059k_{t}+0.002$$ (16)

**Fig 6:**
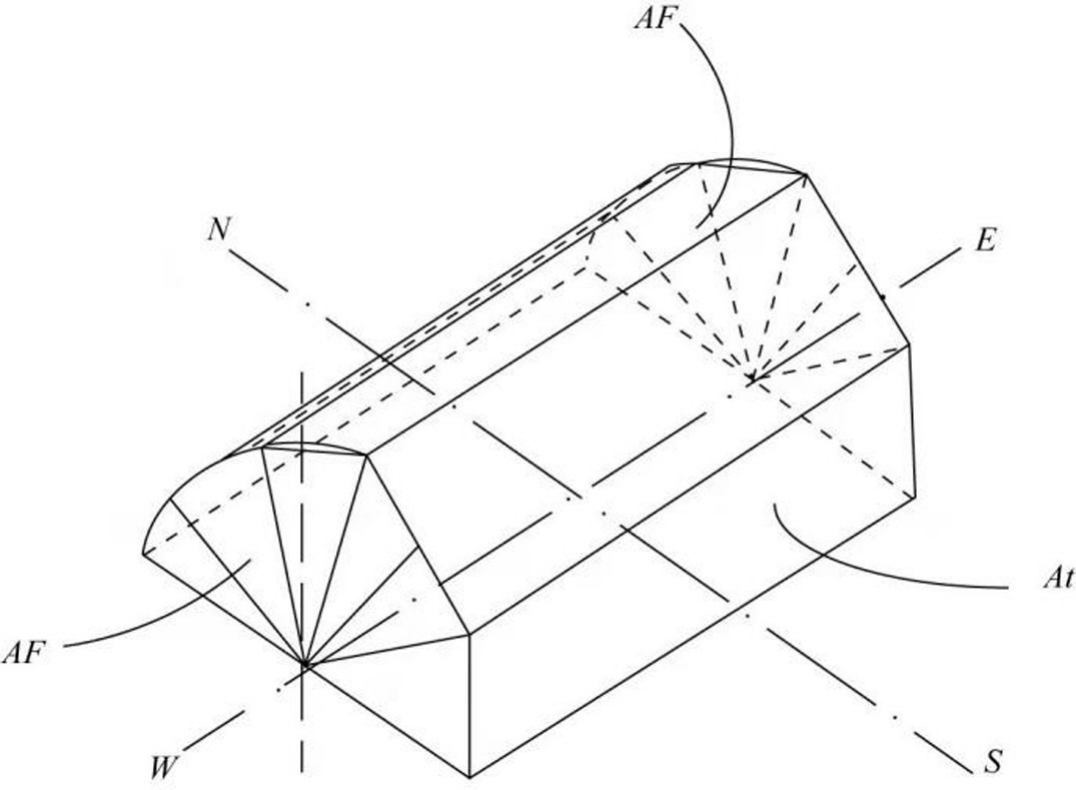
Greenhouse orientation and cover surface.

**Fig 7:**
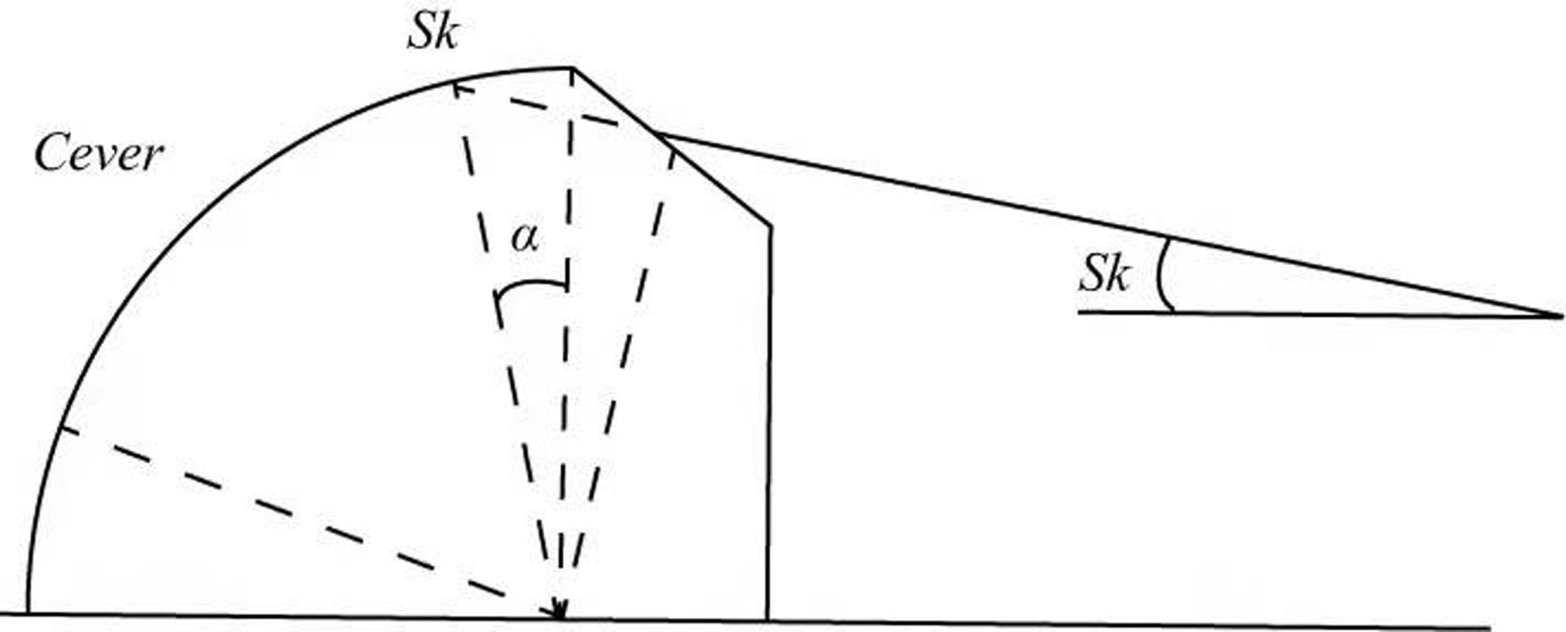
Planar cutting surface of the cover.

**Fig 8:**
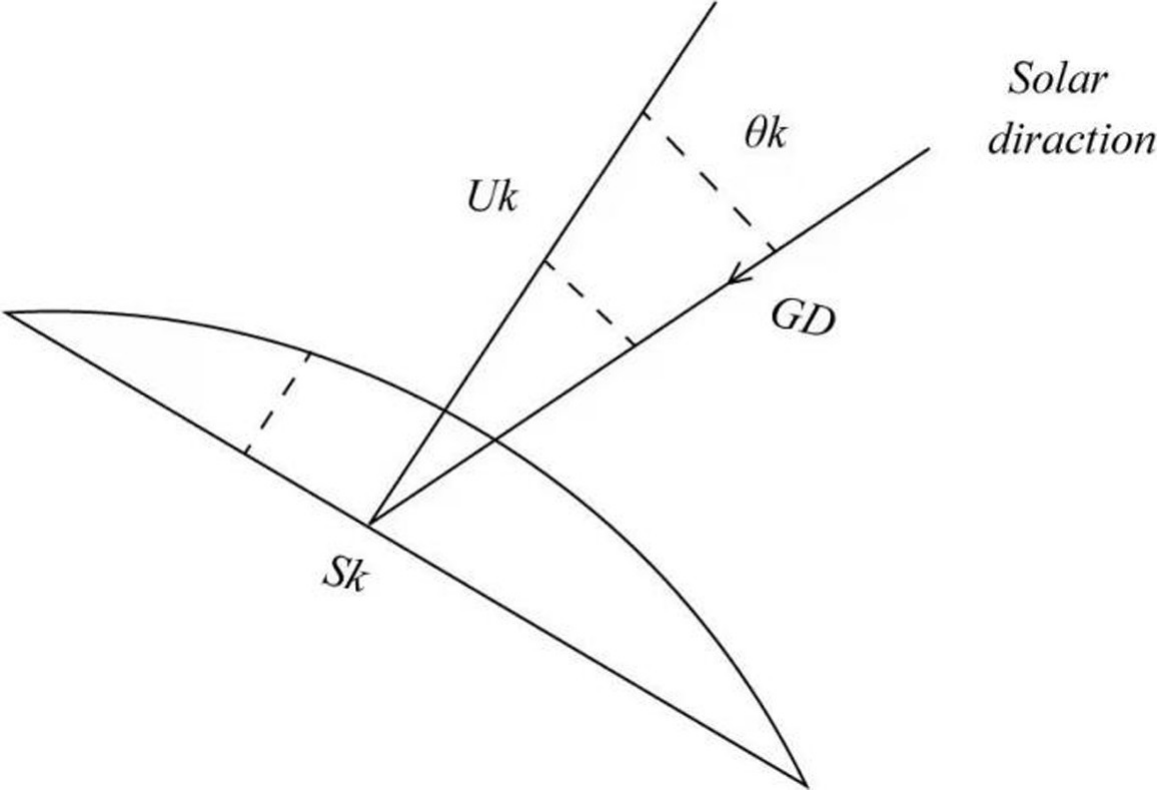
Incident solar radiation angle.

As solar radiation reaches the greenhouse membrane, each part receives a different amount of radiation. Therefore, in order to determine the absorbed radiation, it is very important to determine the total amount of radiation reaching the greenhouse film. As shown in Fig 5, the cover geometry is composed of three planes: the semi-cylindrical east plane, west plane, and upper plane. In order to obtain the optimal estimation of the incident irradiance on the canopy film, the angle between direct solar radiation and the normal of the canopy film must be considered. As shown in Fig 6, when solar radiation is focused on the upper side wall, the cover is cut into *N* equal plane angles. Therefore, as shown in Figs 7 and 8, for each surface angle, the effective radiation flow can be represented as shown in Eq (17):

$$U_{k}S_{k}=G_{D}S_{k}\;cos\;\theta_{k}$$ (17)

The flux of solar radiation received by the upper cover is expressed in Eq (18):

$$\frac{\sum_{K=1}^{N}U_{K}S_{K}}{\sum_{K=1}^{N}S_{K}}=\frac{\sum_{K=1}^{N}G_{D}S_{K}\;cos\;\theta_{K}}{\sum_{K=1}^{N}S_{K}}=G_{D}\frac{\sum_{K=1}^{N}\;cos\;\theta_{K}}{N}$$ (18)

The effective flux of radiation reaching the east wall and the west wall is given by Eqs (19) and (20):

The east wall:

$$G_{E}=\left\{\begin{array}{c} G_{D}\;cos\;\delta \;sin\;w\;w<\frac{\pi }{2}\\ 0\;\text{else} \end{array}\right.$$ (19)

The west wall:

$$G_{W}=\left\{\begin{array}{c} G_{D}\;cos\;\delta \;sin\;w\;w>\frac{\pi }{2}\\ 0\;\text{else} \end{array}\right.$$ (20)

Global (diffuse and direct) outer cover irradiation is now fully represented by Eq (21):

$$E_{ec}=G_{d}+\frac{1}{A_{C}}\left(A_{t}G_{D}\frac{\sum_{k=1}^{N}\;cos\;\theta_{k}}{N}+A_{F}\left(G_{E}+G_{W}\right)\right)$$ (21)

Since the greenhouse axis is set up east-west, the direct sunlight reaching the greenhouse cover described in the references is expressed in Eq (22) [32]:

$$\;cos\;\theta_{k}=\;cos\;\delta \;cos\left(\phi -s_{k}\right)\;cos\;w+sin\;\delta \;sin\left(\phi -s_{k}\right)$$ (22)

If the surface projection on the horizontal plane is southward [37]:

$$s_{k}=\frac{\pi }{2}-\left(k-0.5\right)\mu$$ (23)

If the normal projection on the horizontal plane faces north [37]:

$$s_{k}=\left(k-0.5\right)\mu -\frac{\pi }{2}$$ (24)

Assuming radiation does not propagate across vegetation and soil, the irradiance and irradiance of each surface are represented as follows [38]:

$$E_{s}=j_{ic}F_{sc}+J_{p}F_{sp},E_{p}=J_{s}F_{ps}+J_{pc}+J_{p}F_{pp}$$ (25)

$$E_{ic}=J_{s}F_{cs}+J_{ic}F_{cc}+J_{p}F_{cp}$$ (26)

$$J_{p}=\rho_{p}E_{p},J_{s}=\rho_{s}F_{s}$$ (27)

$$J_{ic}=\rho_{c}E_{ic}+\tau_{c}E_{ec},J_{ec}=\rho_{c}E_{ec}+\tau_{c}E_{ic}$$ (28)

Combining Eqs (25), (26), (27) and (28), radiation can be determined by the matrix expressed in (29):

$$\left(\begin{array}{cccc} 1 & -\rho_{s}F_{sc} & 0 & -\rho_{s}F_{sp}\\ -\rho_{c}F_{cs} & 1-\rho_{c}F_{cc} & 0 & -\rho_{c}F_{cp}\\ -\tau_{c}F_{cs} & -\tau_{c}F_{cc} & 1 & -\tau_{c}F_{cp}\\ -\rho_{p}F_{ps} & -\rho_{p}F_{pc} & 0 & 1-\rho_{p}F_{pp} \end{array}\right)\left(\begin{array}{c} J_{s}\\ J_{ic}\\ J_{ec}\\ J_{p} \end{array}\right)=\left(\begin{array}{c} 0\\ \tau_{c}E_{ec}\\ \tau_{c}E_{ec}\\ \;0 \end{array}\right)$$ (29)

The rear slope of the CSG is mostly a heterogeneous lightweight composite structure. It was assumed that different layers of materials are uniform and divided into M layers according to their thickness. In addition, it was considered that only the rear slope receives solar radiation. The solar radiation received by the surface of the back slope passes through the heat transfer area located between the inner layers. And the solar radiation heat that is transmitted to the innermost layer is represented in Eq (30) [38]:

$$E_{r}=E_{R}a_{R}A_{R}-\sum Cap_{R,M}(M>1)$$ (30)

where *E*_*r*_ is the solar radiation received by the inner surface of the back slope; *E*_*R*_*a*_*R*_*A*_*R*_ is the solar radiation received by the outer surface of the back slope; *E*_*R*_ is the amount of solar radiation; *a*_*R*_ is the solar absorption rate at the outer surface of the back slope; *A*_*R*_ represents the external surface area of the back slope; and Σ*Cap*_*R*,*M*_ is the sum of the heat capacity of all inner layers of the back slope.

The north wall of the CSG is one of the main thermal insulation systems and energy storage structures. It is treated in the same way as the back slope. Therefore, it is divided into N layers along the material thickness. And the thermo-physical properties of each layer are assumed to be consistent. We assumed that only the north wall receives solar radiation. Then the solar radiation received on the inner surface of the north wall is represented in Eq (31):

$$E_{b}=E_{B}a_{B}A_{B}-\sum Cap_{B,N}\text{(}N>1\text{)}$$ (31)

where *E*_*b*_ indicates the solar radiation received by the inner surface of the north wall; *E*_*B*_*a*_*B*_*A*_*B*_ is the solar radiation received by the outer surface of the north wall; *E*_*B*_ corresponds to the amount of solar radiation; *a*_*B*_ is the absorption rate of solar radiation by the outer surface of the north wall; *A*_*B*_ is the area of the outer surface of the north wall; and Σ*Cap*_*B*,*N*_ represents the sum of the heat capacity of each inner layer of the back slope.

Under clear sky conditions, the flux of downward longwave radiation is expressed by Eq (32):

$$q_{cl}=\varepsilon_{cl}^{*}\sigma {\left(e_{o}^{t}\right)}^{4}$$ (32)

where $e_{0}^{t}$ represents the external air temperature (K). On clear day, it is particularly important to find out the empirical expression of the emissivity in the clear atmosphere. Different types (i.e. air temperature, vapor pressure, atmospheric water vapor or perceptible water) and different regression forms (i.e. linear, exponential or quadratic) can provide different models. Since the lower long-wave radiation mainly depends on air temperature and vapor pressure, Eq (33) was expressed as [29]:

$$\varepsilon_{cl}^{*}=0.576{\left(\frac{e_{o}^{v}}{e_{o}^{t}}\right)}^{0.202}$$ (33)

Eq (32) is insufficient to describe the flux of longwave radiation flux during cloudy days. Therefore, an empirical correction was added to the equation. The correction depends largely on cloudy skies. After adjustments, the general expression of longwave radiation flux under all-day conditions is represented in Eqs (34) and (35) [23]:

$$q_{al}=q_{cl}\left(1+Ac^{B}\right)$$ (34)

$$q_{al}=q_{cl}\left(1-c^{D}\right)+Ic^{k}\sigma {\left(e_{o}^{t}\right)}^{4}$$ (35)

where, A, B, D, I and K are adjustment coefficients, and c indicates the cloud cover proportion:

$$c=1-\frac{G_{t}}{G}$$ (36)

where, *G* is the theoretical downward solar radiation on clear sky (Eq 37) [39]:

$$G=G_{0}\;cos\left(\theta_{Z}\right)T_{R}T_{pg}T_{w}T_{a}$$ (37)

where, *G*_0_ is the effective extraterrestrial solar radiation; *θ*_*z*_ is the solar zenith angle; *T*_*w*_, *T*_*pg*_, *T*_*w*_ and *T*_*a*_ represent Rayleigh scattering, absorption of permanent gas, absorption of water vapor, and absorption and scattering transmission coefficient of aerosols, respectively [39].


$$T_{R}T_{pg}=1.021-0.084{\left(\varpi \left(0.000949p+0.051\right)\right)}^{0.5}$$ (38)

$$T_{w}=1-0.077{\left(u\cdot \varpi \right)}^{0.3}$$ (39)

$$T_{a}=0.935^{\varpi }$$ (40)

*ϖ* stands for 0.1013 MPa optical air mass:

$$\varpi =35\;cos\;\theta_{z}{\left(1224{\;cos}^{2}\theta_{z}+1\right)}^{-0.5}$$ (41)

U is precipitation:

$$u=exp\left(-0.073264+0.10146T_{d}-0.0010557T_{d}^{2}\right)$$ (42)

T_d_ is the dew point temperature.

Radiation with heat dissipation depends on temperature of the surface under consideration. The heat flux received by radiation is the difference between the emitted and received irradiance, such as [39]:

$$q_{i}=E_{i}^{*}-J_{i}^{*},$$ (43)

Among them,

$$E_{i}^{*}=\underset{j}{\sum }F_{ji}j_{j}^{*}+H_{0i}^{*}$$ (44)

$$J_{i}^{*}=\sigma \varepsilon_{i}^{*}e_{i}^{4}+\rho_{i}^{*}E_{i}^{*}+\tau_{i}^{*}E_{0i}^{*}$$ (45)

Eq (43) describes the irradiance in the most general case, where *E*_*oi*_ is the transmitted radiation traveling from the outside through the translucent wall; and *H*_*oi*_ is the external radiation of the open system. In thermal radiation modeling, the environment inside and outside the greenhouse must be included. Therefore, the soil, vegetation, and inner wall should be considered in the greenhouse, and the outer wall, outer soil and sky should be included as part of the external environment.

Combined with Eq (43), the generalized radiation equilibrium equation of temperature and heat flow is obtained [39]:

$$-\frac{q_{i}}{\varepsilon_{i}^{*}}-\overset{n}{\underset{j}{\sum }}\left(\frac{1}{\varepsilon_{i}^{*}}-1\right)q_{j}F_{ji}+H_{oi}=\sigma e_{i}^{4}F_{ji}+\frac{\tau_{i}}{\varepsilon_{i}}E_{oi}^{*}-\overset{n}{\underset{j}{\sum }}\frac{\tau_{j}}{\varepsilon_{j}}E_{oj}^{*}F_{ji}$$ (46)

The expression was rewritten as a matrix form and applied to the internal Eq (47) and external environment Eq (48) of the greenhouse to obtain:

$$\left(\begin{array}{ccc} -\frac{1}{\varepsilon_{s}^{*}} & -\frac{\rho_{p}^{*}}{\varepsilon_{p}^{*}}F_{ps} & -\frac{\rho_{c}^{*}}{\varepsilon_{c}^{*}}F_{cs}\\ -\frac{\rho_{s}^{*}}{\varepsilon_{p}^{*}}F_{sp} & -\frac{1}{\varepsilon_{p}^{*}}\left(2-\varepsilon_{p}^{*}F_{pp}\right) & -\frac{\rho_{c}^{*}}{\varepsilon_{c}^{*}}F_{cp}\\ -\frac{\rho_{s}^{*}}{\varepsilon_{s}^{*}}F_{sc} & -\frac{\rho_{p}^{*}}{\varepsilon_{p}^{*}}F_{pc} & -\frac{1}{\varepsilon_{c}^{*}}\left(2-\varepsilon_{c}^{*}F_{cc}\right) \end{array}\right)\left(\begin{array}{c} q_{s}\\ q_{p}\\ q_{ic} \end{array}\right)=\left(\begin{array}{ccc} 1 & -F_{ps} & -F_{cs}\\ -F_{sp} & 1-F_{pp} & -F_{cp}\\ -F_{sc} & -F_{pc} & 1-F_{cc} \end{array}\right)\left(\begin{array}{c} \sigma e_{s}^{4}\\ \sigma e_{p}^{4}\\ \sigma e_{c}^{4} \end{array}\right)+\left(\begin{array}{ccc} 0 & 0 & -\frac{\tau_{c}^{*}}{\varepsilon_{c}^{*}}F_{cs}\\ 0 & 0 & -\frac{\tau_{c}^{*}}{\varepsilon_{c}^{*}}F_{cp}\\ 0 & 0 & \frac{\tau_{c}^{*}}{\varepsilon_{c}^{*}}\left(1-F_{cc}\right) \end{array}\right)\left(\begin{array}{c} 0\\ 0\\ E_{ec}^{*} \end{array}\right)$$ (47)

$$\left(\begin{array}{cc} -\frac{1}{\varepsilon_{ec}^{*}} & -\frac{p_{es}^{*}}{\varepsilon_{es}^{*}}F_{eses}\\ -\frac{p_{ec}^{*}}{\varepsilon_{ec}^{*}}F_{eces} & -\frac{1}{\varepsilon_{es}^{*}} \end{array}\right)\left(\begin{array}{c} q_{ec}\\ q_{es} \end{array}\right)+\left(\begin{array}{c} q_{al}\\ q_{al} \end{array}\right)=\left(\begin{array}{cc} 1 & -F_{esec}\\ -F_{eces} & 1 \end{array}()\left(\begin{array}{c} \sigma e_{ec}^{4}\\ \sigma e_{es}^{4} \end{array}\right)\left(\begin{array}{cc} \frac{\tau_{ec}^{*}}{\varepsilon_{ec}^{*}} & 0\\ \frac{\tau_{ec}^{*}}{\varepsilon_{ec}^{*}}F_{eces} & 0 \end{array}\right)\left(\begin{array}{c} E_{ic}^{*}\\ 0 \end{array}\right)\right)$$ (48)

## Results and discussion

Direct/diffusion separation was replaced by the following formula (16) [40]:

$$\left(\frac{G_{D}}{G_{O}}\right)=\left\{\begin{array}{c} 1-0.09\;k_{t},\;k_{t}\leq 0.22\\ 0.9511-0.1604\;k_{t}+4.388\;k_{t}^{2}-16.638\;k_{t}^{3}+12.366\;k_{t}^{4},\;0.22\leq k_{t}\leq 0.8\\ 0.165\;,\;k_{t}>0.8 \end{array}\right.$$ (49)

Sky temperature was estimated using Eq (50) [41]:

$$e_{\infty }=0.0552e_{0}^{1.5}$$ (50)

The flux of downward longwave radiation expression does not take into account sky emissivity and cloudy sky conditions. Its expression is represented in Eq (51):

$$q_{a}=\sigma e_{\infty }^{4}$$ (51)

The bonding diagram model of CSG was simulated by using 20-SIM software. The numerical solution was obtained through the Adams method [42]. Moreover, data visualization was performed. The direct radiation/diffusion separation equation (i.e. Eq (16)) was used to obtain the instantaneous values of direct radiation and diffusion separation. In addition, Eq (33) was used to simulate longwave radiative heat flows under all-sky conditions. The data introduced by the model included external temperature, external relative humidity, external irradiation, and the number of days and hours. At the beginning of the simulation, all temperatures were set up at the average external temperature of the first day. The parameters of the thermal radiation model and the solar radiation model as well as thermal physical parameters and constants were provided. Because of the small shell thickness, thermal properties were adjusted to 180 μm. In addition, considering the influence of internal and external dust, the optical properties of the greenhouse film were modified.

**Fig 9:**
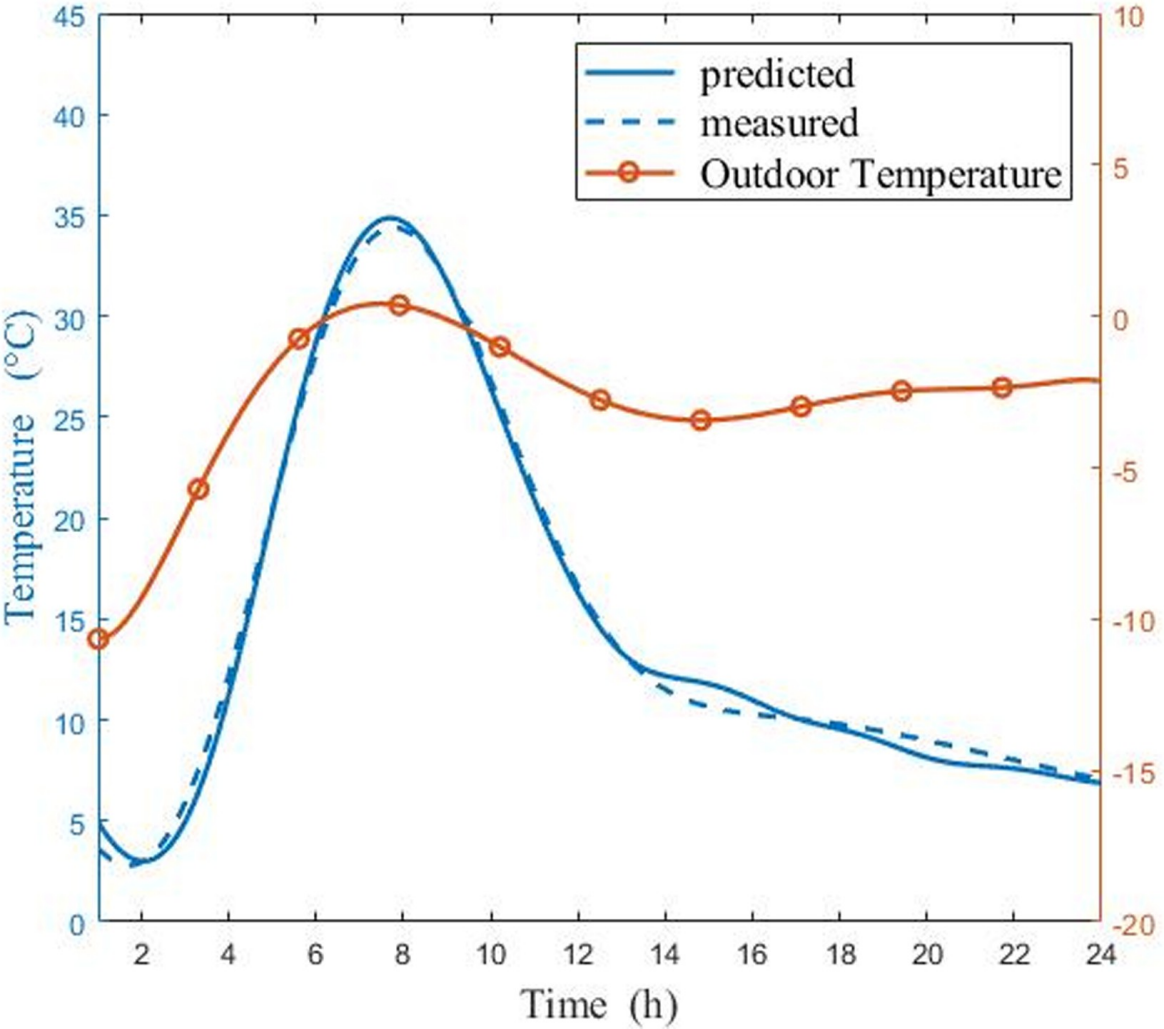
Comparisons of measured and predicted air temperatures in the CSG in sunny day (6:00 in the morning ~ 5:00 the next day).

**Fig 10:**
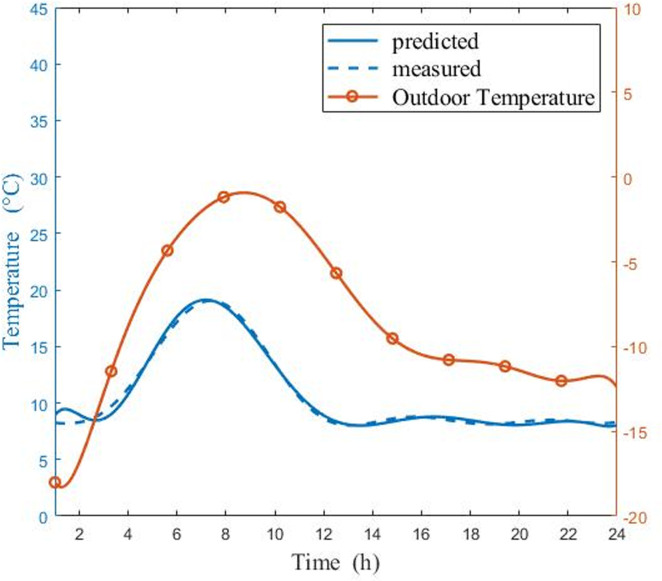
Comparisons of measured and predicted air temperatures in the CSG in cloudy day (6:00 in the morning ~ 5:00 the next day).

Figs 9 and 10 show a comparison of measured and predicted air temperatures in a CSG on sunny and cloudy day, respectively. Fig 9 indicated that, on sunny days, the variation trend of the experimental temperature and the predicted temperature was about the same, and the highest temperature error was 2°C. After a period of t = 7.5 h, with the increase of sun altitude angle, the internal temperature was significantly affected by the temperature rise of outside environment. The sun altitude angle gradually falls over a period of t = 12.5 h (beginning at 12.30 p.m.). The decline in night temperature steadily slowed after a period of t = 20.5 h. on the other hand, the temperature variation, has a multi-peak distribution. Similarly, Fig 10 shows that the measured temperature and the predicted temperature displayed the same trend in cloudy days, and the highest temperature error was about 2°C. The temperature difference between internal and external environment decreases on cloudy day. Compared with the warming rate on sunny day, the warming rate of the CSG slows down on cloudy days. It shows that the tracking condition was acceptable during the early stage of the experiment. Regardless of whether it was a sunny or cloudy day, the same errors were observed at the end of the experiment, which might be caused by external factors. Nevertheless, the overall tracking performance was acceptable. It was also observed that temperature prediction was more accurate before 15:00, and the deviation appeared after this point. These results have proven that the study on radiation refinement significantly improved the model accuracy.

**Fig 11:**
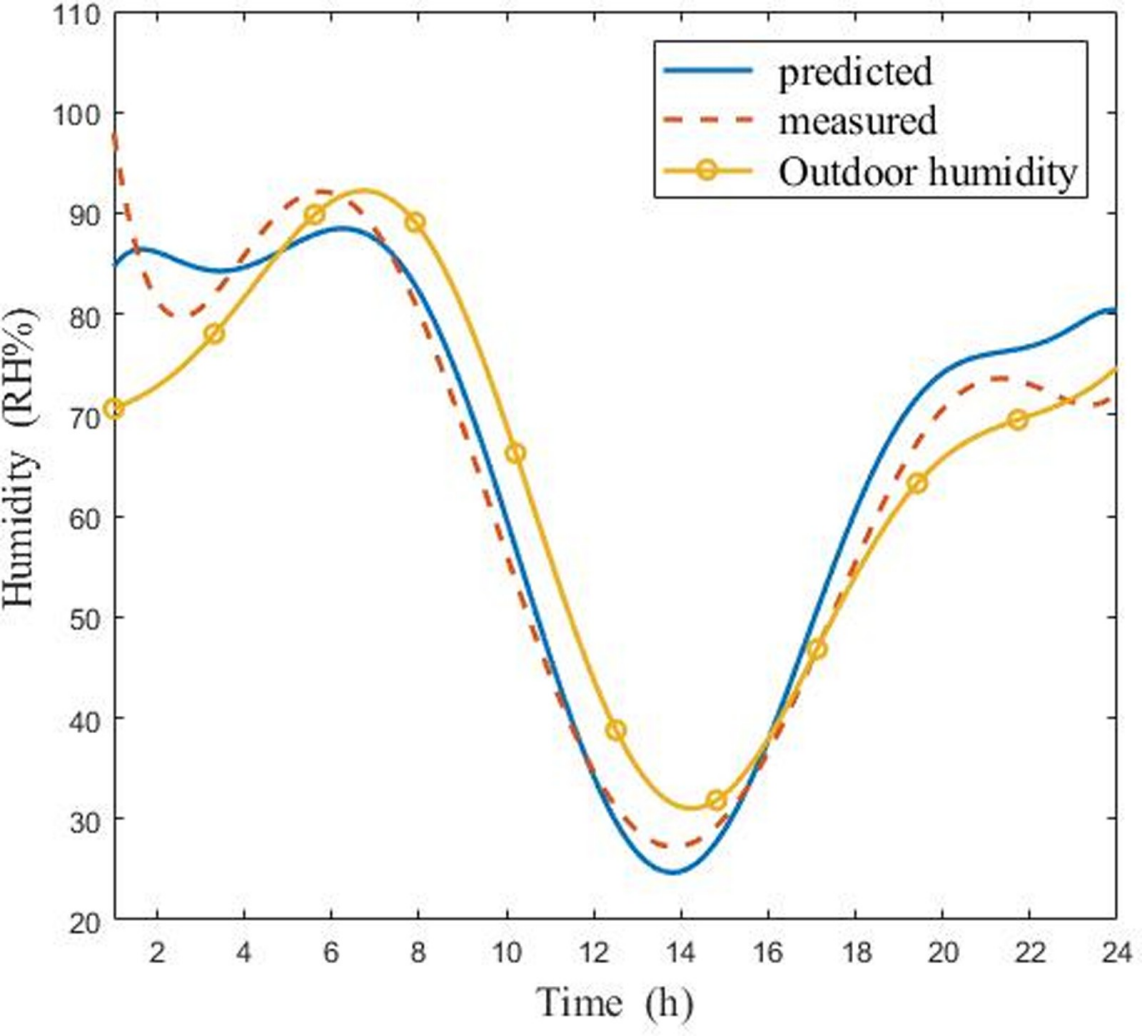
Comparisons of measured and predicted air humidity in CSG in sunny day (6:00 in the morning ~ 5:00 the next day).

**Fig 12:**
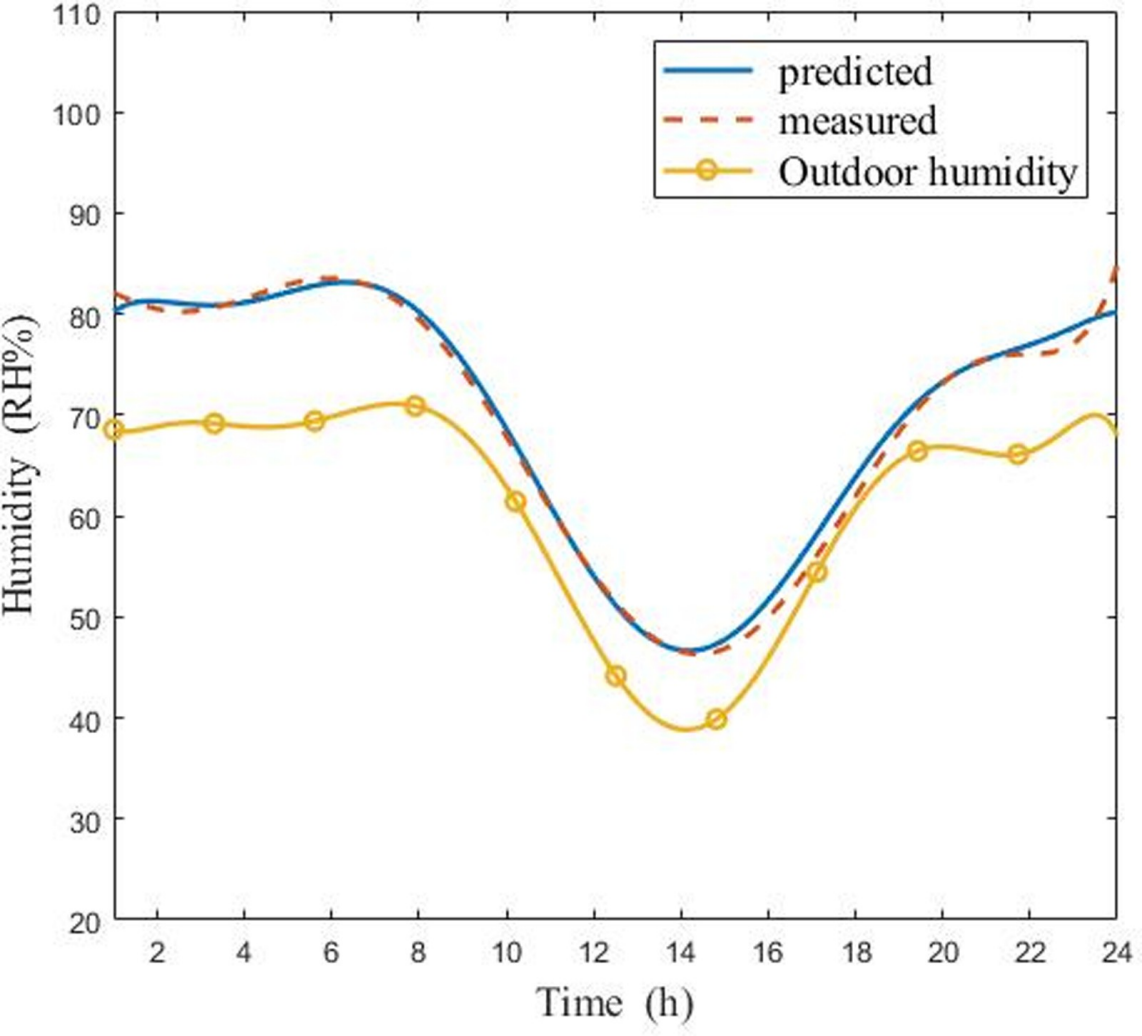
Comparisons of measured and predicted air humidity in CSG in cloudy day (6:00 in the morning ~ 5:00 the next day).

Figs 11 and 12 displayed the comparison of measured and predicted air humidity in the CSG under sunny and cloudy conditions, respectively. As it is shown in Fig 11, the measured humidity and predicted humidity displayed a similar behavior in the sunny weather. Moreover, the highest relative humidity error was 3%. Fig 12 indicated that the variation curves of experimental humidity and predicted humidity were alike, and the maximum relative humidity error was 5% on cloudy days. Despite the temperature has a slight deviation, the results indicated that the overall tracking performance of the curves was in good agreement. On one hand, when the model was proposed, it was assumed that the gas mixture obeyed the ideal gas law, and some details were ignored. On the other hand, the high coupling intensity between temperature and humidity in the greenhouse system may have affected the results. The data obtained in the research showed that the coupling effect of temperature on humidity in the greenhouse was higher than that of humidity on temperature.

The model correctly estimated the temperature and humidity on both sunny days and cloudy days. The simulation results showed that temperature and humidity values in the greenhouse changed greatly. But the variation trend of temperature and humidity value was slightly different. Whether it is sunny or cloudy, the simulation curve of temperature values shows a hump trend of rising first and then falling, however, the simulation curve of humidity values shows a hump trend of falling first and then rising. This clearly showed the importance of Eqs (32)–(42) and the fact that the longwave radiation model takes into account cloudy sky conditions. As seen in Figs 9–12, since energy loss through the greenhouse film was overestimated, the radiation model should to be consolidated when nighttime conditions were considered. The results indicated that simulated temperature and humidity values were in good agreement with the experimental data. It was proved that model displayed high precision and could be used to predict the performance of CSG. By evaluating the entire model, it was found that previous studies underestimated the influence of longwave radiation on temperature and humidity at night, which explained the gap between internal air temperature and relative humidity, and experimental results. The developed model could be a universal and valuable tool that can be used for CSG climate simulation, temperature, and humidity control.

## Conclusion

In the present research, a complete CSG model based on bond graph theory was proposed. Longwave and shortwave radiation were considered in the solar radiation model. The direct and diffusion of shortwave radiation were separately studied. The main conclusions are as follows:

1. The bond graph methodology, which allows *S*_*f*_ elements to be added directly at the field level, suggesting an effective approach to simulate real-world conditions as closely as possible. The effect of longwave and shortwave radiation on the bond graph model was represented by a heat flow in each field.
2. The model was used to predict temperature and relative humidity in the greenhouse on sunny and cloudy days. The predicted temperature showed a trend similar to that of the experimental results. The maximum error of temperature was 2°C and the maximum error of relative humidity was 5%, indicating that the bond graph model could be used to predict the performance of CSG with a high precision.
3. The temperature variation has a multi-peak distribution. After a period of t = 7.5 h, with the increase of sun altitude angle, the internal temperature was significantly affected by the temperature rise of outside environment. The sun altitude angle gradually falls over a period of t = 12.5 h. And the decline in night temperature steadily slowed after t = 20.5 h.
4. The average humidity presents funnel-shaped distribution throughout a day. The maximum value occurs at t = 6.5 h. However, the relative humidity of the internal air begins to descend rapidly with the increase of air temperature, when the blanket quilt covered on the south roof is rolled up. The minimum value occurs at t = 13.5 h.
5. Although some general assumptions have been made, the experimental and the theoretical analysis can improve adaptability and accuracy of the CSG modeling in the future. Meanwhile, some control strategies can be studied on the CSG, such as heated by a buried capillary system and cooled by a mechanical ventilation system.
